# Genomes of diverse Clostridia isolated from a spore forming community in mice that were associated with protection against metabolic syndrome and obesity

**DOI:** 10.1128/mra.00351-24

**Published:** 2024-06-20

**Authors:** Allison M. Weis, Kendra A. Klag, Rickeshia Bell, W. Zac Stephens, June L. Round

**Affiliations:** 1 Department of Pathology, Division of Microbiology and Immunology, University of Utah School of Medicine, Salt Lake City, Utah, USA; Department of Biological Sciences, Wellesley College, Wellesley, Massachusetts, USA

**Keywords:** Clostridia, spore-former

## Abstract

Clostridia are common mammalian gut commensals with emerging roles in human health. Here, we describe 10 Clostridia genomes from a consortium of spore forming bacteria, shown to protect mice from metabolic syndrome. These genomes will provide valuable insight on the beneficial role of spore forming bacteria in the gut.

## ANNOUNCEMENT

Spore forming (SF) bacteria are an important part of a healthy microbiome. Loss of SF bacteria has been associated with diseases including obesity and type 2 diabetes (1–3). Recent literature has found that the class Clostridia provides protection from inflammatory bowel disease, metabolic syndrome, infections, and colorectal cancer (4–7). However, this class of bacteria is often fastidious to grow, which has limited the availability of quality genomes to study.

To advance our understanding of SF bacteria, genomes from 10 isolates were sequenced. To isolate SF bacteria, feces from C57BL/6 specific pathogen-free mice were incubated anaerobically with 0.1% cysteine and 3% chloroform at 37°C for 1 hour to kill off vegetative bacteria. Chloroform was removed by bubbling CO_2_ through the sample for 30 s. To propagate the enrichment of SF bacteria, the sample was gavaged into a breeder pair of germ-free C57BL/6 mice housed in gnotobiotic conditions and feces from resulting offspring were collected, homogenized, serial diluted, and plated on YCFA anaerobically. Individual colonies were picked, streaked to isolation, and liquid cultures started from individual colonies in YCFA; DNA was extracted using Purelink Microbiome DNA purification kit (Invitrogen). Mouse work was performed under IACUC Protocol 00001562.

Five of the isolates' genomes were hybrid assembled from Illumina NovaSeq paired-end 150 and Oxford Nanopore Technologies (ONT) minION reads. Illumina libraries were prepared with NEBNext Ultra II FS DNA kit (NEB, E7805S), and reads were adapter and quality-trimmed with cutadapt (v2.10) (8) in the trim_galore (v0.6.6) wrapper using default parameters. ONT libraries were prepared without DNA shearing or size selection using rapid barcoding kit R9.4.1 chemistry (ONT, SQK-RBK004). Reads were basecalled, demultiplexed, adapter, and barcode-trimmed with guppy (v6.0.1_gpu), then quality-filtered with NanoFilt (9) using a minimum average read quality of 10 and a minimum length of 200. Hybrid genomes were assembled with SPAdes v3.15.5 within Unicycler v0.5.0 pipeline “normal mode” and filtered contigs < 200 bp (10, 11). Five genomes were sequenced and assembled with PacBio reads using Flye v2.8.1 (12) with parameters “--plasmids --iterations 2”. SMRTbell libraries were prepared without shearing using PacBio Express Template Prep Kit 2.0, pooled, and size selected using Sage Sciences' BluePippin with 0.75% DF Marker S1 High-Pass 6–10 kb v3 run protocol, S1 marker, and a cutoff of 8,000 (BPstart value), and then libraries were bound per SMRT Link Setup and sequenced on a Sequel II. Assemblies were annotated by NCBI's PGAP v6.6 (13).

All isolates are domain Bacteria, phylum Bacillota, class Clostridia, and order Eubacteriales (see Table 1 for full NCBI-assigned taxonomy). While some of the genomes had published close matching reference genomes, others such as *Lachnospiraceae* KK002 and KK008 were as far away as 88% and 78% best match by average nucleotide identity (ANI), respectively (FastANI v0.1.3 via GTDB-tk toolkit), indicating that they are likely new and undescribed species (14). Each genome's features including GC% content, genome size, and the number of predicted genes are described in Table 1.

**TABLE 1:** Taxonomy and genome characteristics of spore forming bacteria^*a*^

Isolate ID	GenBank accession	Taxon beyond class clostridia order eubacteriales	Closest ref genome	ANI	Sequencing	Contigs	N50	Size	%GC	CDS	SRR	Illumina Paired-End read number	PacBio subread number	PacBio subread N50	ONT read number	ONT read N50	ONT flowcell
JLR.KK001	JAYMNO000000000	*f__Lachnospiraceae;* *g__Sporofaciens sp. KK001*	GCA_9105 74715.1	98.44	Hybrid	31	4,968,565	6,390,410	46%	6,399	SRR28014014, SRR28014007	4,717,052	NA	NA	26,547	7,327	FLO-MIN106
JLR.KK005	JAYMNR000000000	*f__Candidatus;* *g__Ventrimonas sp. KK005*	GCA_0099 11065.1	96.15	Hybrid	205	197,068	5,083,539	44%	4,949	SRR28014013, SRR28014006	3,632,234	NA	NA	15	4,054	FLO-FLG001
JLR.KK006	JAYMNS000000000	*f__Lachnospiraceae;* *g__Candidatus Merdisoma sp. KK006*	GCA_9105 74255.1	98.86	Hybrid	112	111,685	4,698,239	45%	4,502	SRR28014010, SRR28014005	5,151,028	NA	NA	77	3,513	FLO-FLG001
JLR.KK011	JAYWSZ000000000	*f__Lachnospiraceae;* *g__Candidatus Merdisoma sp. KK011*	GCA_9105 75725.1	98.57	PacBio	7	2,532,420	5,043,844	48%	4,958	SRR28516891	NA	92,862	11,648	NA	NA	NA
JLR.KK002	JAYMNP000000000	*f__Lachnospiraceae bacterium KK002*	GCF_0004 03845.2	88.4	Hybrid	5	4,212,295	4,276,026	46%	4,102	SRR28014009, SRR28014004	4,880,942	NA	NA	11,608	8,894	FLO-MIN106
JLR.KK008	CP143548	*f__Lachnospiraceae bacterium KK008*	GCA_9105 85345.1	78.35	PacBio	1	3,188,748	3,188,748	48%	3,078	SRR28516890	NA	39,804	12,282	NA	NA	NA
JLR.KK009	CP143549	*f__Lachnospiraceae bacterium KK009*	GCA_0004 03315.2	99.31	PacBio	1	5,268,209	5,268,209	47%	5,165	SRR28516889	NA	81,055	12,085	NA	NA	NA
JLR.KK004	JAYMNQ000000000	*f__Oscillospiraceae;* *g__Acutalibacter sp. KK004*	GCF_0099 36035.1	96.44	Hybrid	12	2,635,137	3,852,823	54%	4,046	SRR28014008, SRR28014003	3,640,502	NA	NA	15,286	8,990	FLO-MIN106
JLR.KK007	JAYWSY000000000	*f__Oscillospiraceae;* *g__Lawsonibacter sp. KK007*	GCA_9105 84605.1	98.48	PacBio	5	1,637,602	4,295,262	58%	4,458	SRR28516888	NA	109,452	12,756	NA	NA	NA
JLR.KK010	CP143550	*f__Eubacteriales Family XIII;g__Emergencia sp. KK010*	GCF_0099 36045.1	98.09	PacBio	1	3,076,573	3,076,573	44%	2,874	SRR28516887	NA	260,145	12,915	NA	NA	NA

^*a*^ NA = Not applicable.

## Data Availability

All sequences are available through NCBI Bioproject PRJNA1061597. GenBank and SRA accession numbers listed in Table 1. Isolates are available from the Round Lab.
